# Supplementary material for: The interactome of intact mitochondria by cross-linking mass spectrometry provides evidence for coexisting respiratory supercomplexes
Source: Mol Cell Proteomics. 2017 Dec 8;17(2):216–32. doi: 10.1074/mcp.RA117.000470 (PMC5795388; doi:10.1074/mcp.RA117.000470)
Supplement: Supplemental Data [file supp_RA117.000470_133922_0_supp_23978_fzffwf.zip › spectra_annotation/mito_spectra_annotation/433-1-1-1-12-1.pdf]

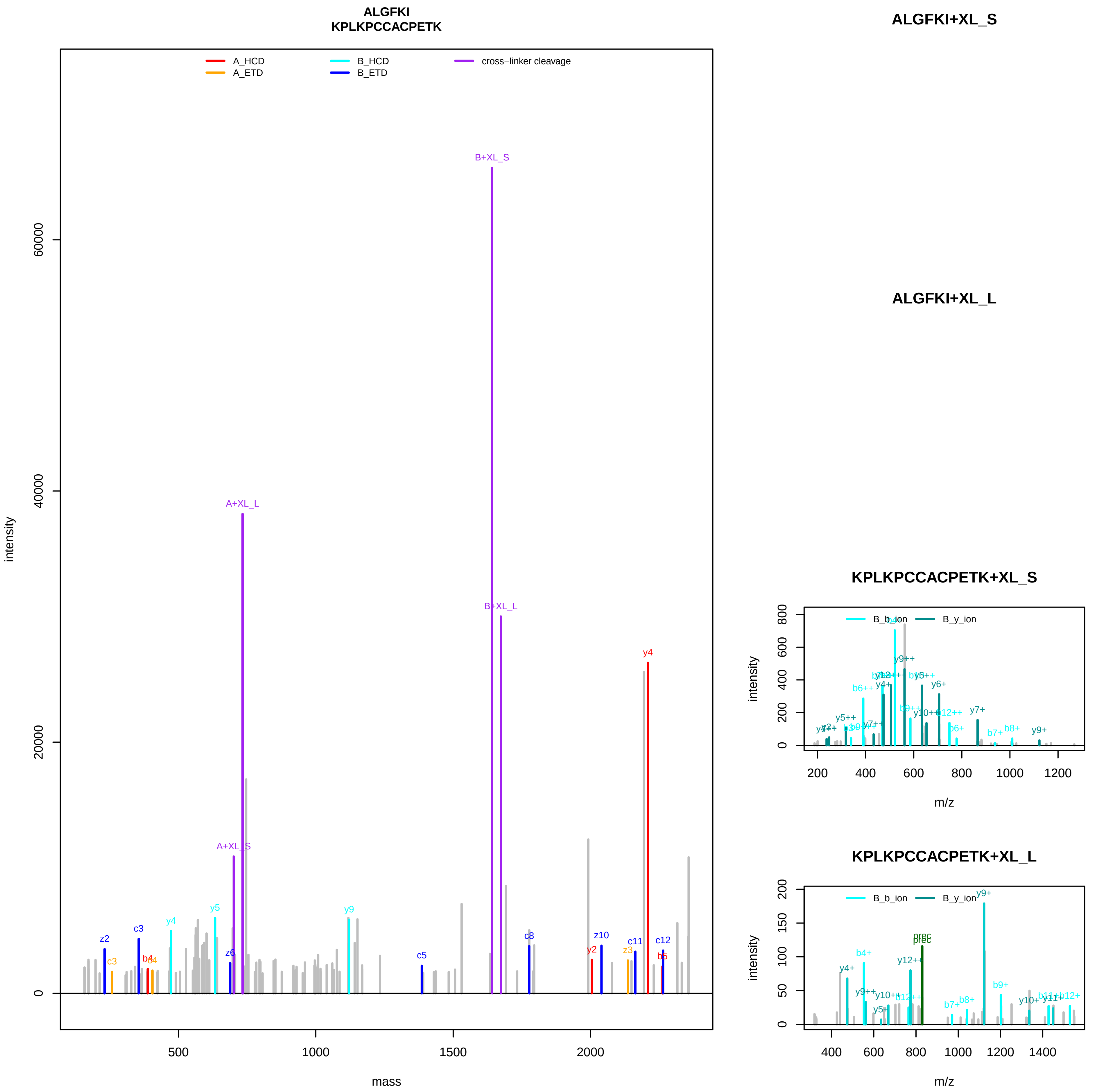
ALGFKI
KPLKPCCACPETK
A_HCD
A_ETD
B_HCD
B_ETD
cross−linker cleavage
B+XL_S
A+XL_L
B+XL_L
A+XL_S
y4
z2
c3
c3
b4
c4
y4
y5
z6
y9
c5
c8
y2
z10
z3
c11
c12
b5
0
20000
40000
60000
intensity
500
1000
1500
2000
mass
ALGFKI+XL_S
ALGFKI+XL_L
KPLKPCCACPETK+XL_S
B_b_ion
B_y_ion
intensity
m/z
KPLKPCCACPETK+XL_L
B_b_ion
B_y_ion
prec
intensity
m/z
